# Exchange of functional domains between a bacterial conjugative relaxase and the integrase of the human adeno-associated virus

**DOI:** 10.1371/journal.pone.0200841

**Published:** 2018-07-17

**Authors:** Leticia Agúndez, Francisco Zárate-Pérez, Anita F. Meier, Martino Bardelli, Matxalen Llosa, Carlos R. Escalante, R. Michael Linden, Els Henckaerts

**Affiliations:** 1 Department of Infectious Diseases, School of Immunology and Microbial Sciences, King's College London, London, United Kingdom; 2 Department of Physiology and Biophysics, Virginia Commonwealth University School of Medicine, Richmond, Virginia, United States of America; 3 Instituto de Biomedicina y Biotecnología de Cantabria (IBBTEC), Universidad de Cantabria-CSIC-SODERCAN, Santander, Spain; Centre National de la Recherche Scientifique, Aix-Marseille Université, FRANCE

## Abstract

Endonucleases of the HUH family are specialized in processing single-stranded DNA in a variety of evolutionarily highly conserved biological processes related to mobile genetic elements. They share a structurally defined catalytic domain for site-specific nicking and strand-transfer reactions, which is often linked to the activities of additional functional domains, contributing to their overall versatility. To assess if these HUH domains could be interchanged, we created a chimeric protein from two distantly related HUH endonucleases, containing the N-terminal HUH domain of the bacterial conjugative relaxase TrwC and the C-terminal DNA helicase domain of the human adeno-associated virus (AAV) replicase and site-specific integrase. The purified chimeric protein retained oligomerization properties and DNA helicase activities similar to Rep68, while its DNA binding specificity and cleaving-joining activity at *oriT* was similar to TrwC. Interestingly, the chimeric protein could catalyse site-specific integration in bacteria with an efficiency comparable to that of TrwC, while the HUH domain of TrwC alone was unable to catalyze this reaction, implying that the Rep68 C-terminal helicase domain is complementing the TrwC HUH domain to achieve site-specific integration into TrwC targets in bacteria. Our results illustrate how HUH domains could have acquired through evolution other domains in order to attain new roles, contributing to the functional flexibility observed in this protein superfamily.

## Introduction

The HUH (His-hydrophobic-His) superfamily of endonucleases is specialized in processing single-stranded (ss) DNA through site-specific recognition of the target site, cleavage and strand-transfer reactions [1]. HUH endonucleases can be found in all three domains of life, and their biological relevance is mostly related to their ability to process mobile genetic elements. They were first classified by Ilyna and Koonin based on their highly conserved motifs: a HUH motif required for metal ion binding, and a motif composed by one or two catalytic tyrosines (Tyr(s) motif) [2]. A common evolutionary origin was proposed for the superfamily [2, 3], a proposal which was later reinforced by the striking structural similarities among members of the different subfamilies [4]. Within this superfamily, members are classified into 3 groups: (i) rolling-circle replication proteins (RCR or Rep proteins), (ii) conjugative plasmid transferases (relaxases) or Mob (mobilization) proteins, and (iii) DNA transposases. Rep proteins are responsible for initiation and termination of rolling-circle replication (RCR), a mechanism adopted by many bacteriophages (e.g. ϕX174) [5], eukaryotic viruses (e.g. adeno-associated virus (AAV), TYLCV, circoviruses) [6, 7] and bacterial plasmids (e.g. RepB-pMV158) [8]. Rep initiator proteins nick one strand of the substrate DNA at the origin of replication (*oriV*) to provide a free 3’-OH end onto which nucleotides are added, while the other strand is displaced from the ds circle and copied [9]. Bacterial conjugative relaxases nick one strand at the origin of transfer (*oriT*) to pilot the ssDNA from a donor to a recipient bacterium through a protein channel named Type IV secretion system (T4SS) [10]. As in RCR, the relaxase is responsible for termination of the DNA transfer process through a strand-transfer reaction that takes place in the recipient cell [11]. RCR transposases mediate a transposition mechanism that resembles biochemically the aforementioned initiation and termination reactions, but includes the formation of a circular single-stranded transposon as an intermediate step [12]. The similarity of the reactions catalysed by HUH proteins involved in these different biological processes is further underlined by the finding that certain members of one subfamily perform the biological role of others, reinforcing their evolutionary origin. For example, some conjugative relaxases are also involved in the initiation of replication of their cognate mobile elements [13], while some Rep proteins can mobilize DNA for conjugal transfer [14].

Several authors have proposed that the functional versatility of the HUH superfamily is attained in part by modular evolution [1, 15], through the addition of further domains, such as ATP-dependent DNA helicase domains, Zn-finger binding domains, primase and oligomerisation domains, and/or by recruiting host cell proteins. These acquired functions can go beyond the canonical HUH roles in replication, conjugation or transposition. For example, HUH endonucleases such as AAV-Rep78/68 [16], the relaxase TrwC [17], and the transposase TnpA (REP) [18] support site-specific recombination (SSR) and integration (SSI) of DNA substrates with the aid of the host replication machinery and have been proposed as a new family of site-specific recombinases [19].

The AAV replication proteins Rep78 and Rep68 are multi-domain proteins involved in all aspects of viral replication. They display DNA binding, endonuclease and helicase activities [20–22] and are required for regulation of transcription [23] and DNA replication [24, 25] of this helper virus-dependent single-stranded human DNA virus. Moreover, in the absence of helper virus infection, Rep78/68 proteins can promote site-specific integration (SSI) of the viral genome into specific loci in the human genome [26–28]. The N-terminal origin binding domain (OBD, residues 1–208) of Rep78/68 contains the HUH and the Tyr(s) motifs characteristic of HUH endonucleases. It recognises the Rep binding site (RBS) and the nicking site, also known as terminal resolution site (*trs*), which are present in the viral inverted terminal repeats (ITR) as well as in the chromosomal integration sites, and exhibits site-specific endonuclease activity [29, 30]. The OBD of Rep78/68 constitutes a catalytic domain sufficient to mediate site-specific endonuclease activity [29]. The central domain is an AAA+ ATPase and is characteristic of the superfamily 3 (SF3) of helicases [31]. The C-terminal domain of Rep78, which is not present in the alternative splice variant Rep68, is a zinc finger domain, involved in dimerization and protein-protein interactions [32, 33]. Recently, the linker connecting the OBD and helicase domains, comprising residues 215–224, has been shown to be required for functional oligomerization and cooperative action of the OBD and the helicase domain [34, 35]. The oligomeric behaviour of AAV Rep78/68 is complex and dynamic, and contributes to the extraordinary multi-functionality of these viral proteins [36, 37].

The relaxase TrwC of the conjugative plasmid R388 belongs to the HUH subgroup of proteins responsible for DNA processing during bacterial conjugation. In the donor cell, it processes the single strand to be transferred, leading it into a recipient bacterium, where it catalyses the final recircularization step. TrwC possesses DNA binding, endonuclease and DNA helicase activities. The N-terminal 298 residues of the protein retain full relaxase activity [38], including recognition of the target DNA sequence *oriTw*, which contains an inverted repeat (IR) acting as a binding site, and cut-and-paste activities on a nicking site (*nic*), catalysed by two Tyr residues [38–40]. The C-terminal domain of TrwC is an SF1 helicase, which mediates ATP hydrolysis, DNA unwinding, and dimerization [41]. In addition to its role in conjugation, TrwC can act as a site-specific recombinase and integrase [17, 42]. Specifically, it can catalyse integration of the incoming plasmid DNA into a target sequence present in the recipient bacterial chromosome or plasmid [17, 43], in a reaction requiring both catalytic tyrosines [43]. These reactions cannot be performed by the relaxase domain alone, requiring the N-terminal 450 residues of TrwC [43]. However, DNA unwinding activity, which is essential for conjugation, is dispensable for SSI [43].

In summary, AAV-Rep78/68 and R388-TrwC share biochemical as well as functional properties, including the intriguing ability to mediate site-specific integration. Both proteins perform the nicking reaction required for integration by a similar mechanism: DNA interrogation, high affinity binding and unwinding, and local origin melting, which cause an extrusion of the nicking site, allowing the protein to engage specifically with ssDNA [38, 44]. Despite these similarities, there are some intrinsic differences, such as the number of catalytic Tyr residues (2 in TrwC, 1 in Rep78/68) [11, 40, 45], and the oligomeric state of the proteins [36–38, 46}, which might be reflective of the differences in life cycle of bacterial plasmid and mammalian virus.

Given the overall similarities between the AAV-Rep78/68 and R388-TrwC HUH domains and their different biological functions, we considered them a suitable target to test the hypothesis that this functional HUH unit for the nicking and re-ligation activities could be exchanged between distantly related proteins. We have engineered a TrwC/Rep chimeric protein consisting of the N-terminal TrwC relaxase domain and the C-terminal AAV Rep68 helicase domain, and performed a variety of biochemical and biophysical analyses to investigate whether exchange of the HUH endonuclease domains of these proteins is tolerated. Moreover, we investigated the ability of the chimeric protein to mediate site-specific integration in bacterial and human cells. These studies reveal that endonuclease domain exchange maintains the functionality of each domain at the same level as the parental proteins, while cross-talk between domains of different parental origin allows the chimeric protein to execute additional functions. These results support the biochemical conservation between the distantly related HUH domains, and provide an example on how HUH proteins may have attained such diverse biological roles.

## Materials and methods

### Plasmids

Plasmids used are described in Table 1. Plasmids were generated using standard cloning techniques. *Escherichia coli* strain DH5α [47] was used for plasmid construction and maintenance. Restriction enzymes, calf intestinal alkaline phosphatase and T4 DNA ligase were purchased from NEB. PCRs were performed using Phusion high fidelity DNA polymerase (NEB). All newly generated plasmids were verified by DNA sequencing.

**Table 1:** Plasmids used in this work.

Plasmid	Description	Reference
p220.2	EBV-based shuttle vector	[48]
p220.2::*AAVS1*(kb 0–0.51)	Contains AAVS1 minimal target site for AAV integration	[49]
pCEFL	Eukaryotic expression vector	[50]
pCMS11	oriV(R6K)::*RP4 oriT +R388 oriT + nptII*	[17]
pCMV::*rep68*	*rep68* driven by CMV promoter	[51]
pDsRedN1	Cloning vector	Clontech
pET15b	Cloning vector	Novagene
pET15b::*PsP rep68*	His Tag and Precision protease site Rep68	[37]
pIRES-eGFP	Vector expressing EGFP	Clontech
pLA14	pCEFL::*trwC*	[52]
pLA32	pSU36:: *oriTw*	[43]
pLA58	p220.2::*oriTw* nick antisense	This study*
pLA59	p220.2::*oriTw* nick sense	This study*
pLA106	pET15b::*PsP trwC-rep68 chimera*	This study*
pLA107	pCEFL::*trwC-rep68 chimera*	This study*
pLA117	pDsRedN1:: *trwC-rep68 chimera*	This study*
pLA119	pDsRedN1:: *trwC*	This study*
pLA131	pET3a::*trwC-rep68 chimera*	This study*
pRVK	Contains *AAVS1* nucleotide 1 to 3536 bp	[53]
pSU1186	pUC18::*oriTw*	[54]
pSU1600	pET3a::*trwC*(N293)	[42]
pSU1621	pET3a::*trwC*	[38]
pSU2007	Plasmid R388 KmR derivative. Contains *oriTw*	[55]
pTRUF11	Contains hGFP and NeoR, flanked by AAV2 ITRs	[56]

*See Materials and Methods for details on plasmid construction.

The plasmid expressing the *trwC/rep* chimera gene, named pLA106, was obtained by overlapping PCR as follows. Two separate products were amplified using primers A (5’—CCAACATATGctggaagttctgttccaggggccc**CTCAGTCACATGGTATTGA** -3’) and A’ (5’- *GGCGCATCAGAATTGGG***GCTGAAATCTATGCCGAG** -3’) on pLA14 as template (Table 1), and primers B (5’-**CTCGGCATAGATTTCAGC***CCCAATTCTGATGCGCC* -3’) and B’ (5’- CCAACTCGAG*TCAGAGAGAGTGTCCTCGA-*3’) on pET15b::*PsP rep68* (Table 1) as template. The sequence corresponding to *rep68* or *trwC* is indicated in italics and boldface, respectively. Primer A contains the PreScission Protease site (PsP, lowercase) after the *Nde*I site (which contains the ATG start codon), followed by nt 4 to 22 of *trwC*. In primer A’, nt 1–17 correspond to nt 625–641 of *rep68*, and nt 18–35 to 862–879 of *trwC*. In primer B, nt 1–18 correspond to 862–879 of *trwC* and nt 19–35 correspond to nt 625–641 of *rep68*. Primer B’ contains an *Xho*I site followed by nucleotides coding the end of *rep68*. The resulting PCR products were gel purified, combined and used as templates for an overlapping PCR with primers A and B’. The product of 1.9 kb was digested with *Nde*I and *Xho*I and ligated to pET15b containing the His tag for protein purification, which was treated with the same enzymes and gel purified. For immunofluorescence assays, the *trwC/rep* chimera sequence was amplified from pLA106 with primers 5’-CCAAGATATC**ATGCTCAGTCACATGGTATTGAC-3’** and 5’-CCAAGCGGCCGC*TCAGAGAGAGTGTCCTCGA*-3’, and the introduced *Eco*RV and *Not*I sites (underlined) were used for cloning into the same sites of the pCEFL vector under the control of the eukaryotic promoter elongation factor 1α (EF1α), resulting in plasmid pLA107. For site-specific integration in human cells, the *oriTw* nick antisense fragment was amplified from plasmid pSU2007 with primers 5’-CCATCTAGACTCATTTTCTGCATCATTGT-3’ and 5’-AACAAGCTTCCTCTCCCGTAGTGTTAC-3’, introducing *Xba*I and *Hind*III (underlined) sites to clone into p220.2 generating pLA58. For pLA59, *oriTw* nick sense, the strategy was as described before, but primers 5’- CCATCTAGACCTCTCCCGTAGTGTTAC-3’ and 5’-AACAAGCTTCTCATTTTCTGCATCATTGT-3’ were used. To express the chimeric protein in human cells for the episomal assay, pLA117 was generated by subcloning a *Kpn*I/*Not*I fragment from pLA107 into pDsRedN1. pLA119, to test TrwC protein in the episomal assay, was obtained by amplifying *trwC* from plasmid pLA14 and primers 5’- CCAGGTACCATGCTCAGTCACATGGTATTGAC-3’ and 5’-CCAAGCGGCCGCCTTACCTTCCGGCCTCCA-3’. The introduced *Kpn*I/*Not*I sites (underlined) were used to clone into the same sites in pDsRedN1. The construct pLA131, used to test the integration ability of the chimeric protein in bacteria, was generated by subcloning an *Nsi*I/*Cla*I fragment from pLA106 into pSU1621 (Table 1).

### Protein purification

*Rep68* and *trwC/rep chimera* cloned into the pET15b vector were expressed in *E*. *coli* BL21(DE3) carrying pLysS (Stratagene). Rep68 His-tagged protein was isolated as follows. 1l of culture was induced with 1mM IPTG for around 2 h at 37°C until OD600 reached 0.5–0.6, pelleted and kept at -80°C. Pellets were then resuspended in 20 ml Ni column buffer A (20 mM Tris HCl, 500 mM NaCl, 5 mM Imidazole, 10% Glycerol and 0.05% NP-40) at pH 7.9 and 20 ml BPER buffer (Pierce), plus 2 μg/ml aprotinin, 2 μg/ml leupeptin, 1 μg/ml pepstatin, and 600 μM PMSF. The lysate was sonicated, spun, filtered and loaded onto a 5 ml nickel affinity column (GE Healthcare) and washed with increasing imidazole concentrations. His-Rep68 was eluted in buffer B (Buffer A and 300 mM imidazole). The eluate was then loaded onto a gel filtration column HiPrep 16/60 Sephacryl S200 HR (GE Healthcare), which was equilibrated in protein storage buffer (25 mM Tris HCl, 600 mM NaCl, 5% Glycerol and 1 mM TCEP) at pH 7.6. The protein was concentrated to 1.0 mg/ml, aliquoted and stored at -80°C. The His-TrwC/Rep chimera was purified following the same protocol, but was eluted in buffer containing 150 mM imidazole.

For analytical centrifugation and fluorescence anisotropy assays, the chimeric protein was purified from the affinity column equilibrated with buffer A’ (20 mM Tris HCl, 200 mM NaCl, 5 mM imidazole, 10% Glycerol, 2% CHAPS and 1mM TCEP) at pH 7.9 and eluted as described above. Then, it was desalted using buffer A’ and a HiPrepTM 26/10 desalting column (GE Healthcare). His-PsP tag was removed by PreScission protease (PsP) treatment using 150 μg/mg of protein. After overnight incubation at 4°C, buffer was exchanged using the same desalting column and Ni-Buffer A’. Subsequent Ni column chromatography using the buffer B’ (same as buffer A’ but with 1 M imidazole) was performed to remove the uncleaved fusion protein, and untagged protein was eluted. The TrwC/Rep chimera protein was finally purified by gel filtration chromatography equilibrated in protein storage buffer (25 mM Tris-HCl, 200 mM NaCl and 1 mM TCEP) at pH 7.9.

### Western blots

Transient transfection of 293T cells was performed in 6-well plates using 5x10^5^ cells per well. At 70% confluence, cells were transfected with 4 μg of either a GFP-expressing plasmid (pTRUF11, Table 1) as a transfection control, pCEFL (negative control), pCMV::*rep68*, or pLA107 (pCEFL::*trwC/rep chimera*) and 10 μl of Lipofectamine 2000 according to the manufacturer’s instructions (Invitrogen). 48 h post transfection, cells were harvested and resuspended in 600 μl of 2X Laemmli buffer, boiled for 5 min and centrifuged at 3000 rpm for 10 min. 20 μl of cleared supernatant was run on a 12% SDS-PAGE gel and transferred to a nitrocellulose membrane (Hybond-C Extra nitrocellulose; Amersham Biosciences). Membranes were blocked in 10 ml of 0.1% PBS-Tween with 5% non-fat dry milk for 1 h and incubated with primary antibody for 3 h at room temperature. Antibodies used were α-Rep monoclonal antibody directed against the C-terminal domain, clone 226–7 from Acris Antibodies, catalog no. BM5012SU (1:100 in 0.1% PBS-Tween with 5% non-fat dry milk) and α- β actin mouse monoclonal antibody (1:500 in 0.1% PBS-Tween with 5% non-fat dry milk) from BD Biosciences, catalog no. 612656. The membranes were washed 3 times, 10 min each in 10 ml 0.1% PBS-Tween and then incubated with goat anti-mouse (Jackson ImmunoResearch Laboratories; catalog no. 115-035-003) secondary antibody conjugated to horseradish peroxidase used at a dilution of 1: 10,000 in 10 ml of 0.1% PBS-Tween with 5% non-fat dry milk for 1 h at room temperature. After 3 washes, the membranes were developed using enhanced-chemiluminescence (ECL) substrate (Pico detection kit; Pierce) and scanned using ImageQuant (GE Healthcare Life Sciences).

### Immunofluorescence assays

293T cells were maintained in DMEM supplemented with 10% FBS. A total of 3x10^5^ cells were seeded in 60 mm plates in 5 ml medium on glass coverslips and grown for 48 h. Cells were transfected using 4 μg of either pTRUF11 (to control transfection), pCEFL (as negative control), pCMV::*rep68*, pLA14 (pCEFL::*trwC*) or pLA107 (pCEFL::*trwC/rep* chimera) and 10 μl of Lipofectamine 2000. After 30h, cells were fixed and permeabilized with methanol for 10 min at -20°C. Cells were incubated with either primary α-TrwC [46] or α-Rep monoclonal antibody clone 226–7 (1:50 dilution in PBS) for 3 h at room temperature, washed in PBS, and incubated with secondary fluorescein isothiocyanate-conjugate goat anti-mouse antibody (1:500 dilution in PBS; Jackson laboratories) for 1 h at room temperature. Coverslips were mounted in Vectashield Mounting Medium containing DAPI (4’,6-diamidino-2-phenylindole; Vector Laboratories). Images were acquired at 60x magnification using a Leica DMRA2 fluorescence microscope with a Hamamatsu CCD digital camera and analyzed with Openlab software (Improvision).

### DNA helicase assay

DNA unwinding was assessed using an M13-based partially duplex substrate as described previously [57]. The M13(-20) primer was first annealed to M13mp18 DNA (NEB), followed by 3’ extension by Klenow DNA polymerase (NEB) in the presence of dGTP and α-[32P]-dCTP (6,000 Ci/mmol; Perkin Elmer). After labelling, the sample was passed through a Sephadex illustra Microspin G50 column (GE Healthcare). For the reaction, different amounts of purified His-Rep68 or His-TrwC/Rep chimeric proteins were incubated with 100 fmol radiolabeled substrate, 20 mM Tris HCl (pH 7.5), 20 mM MgCl2, 8 mM DTT, 5 μg/ml BSA, 4% sucrose, in the presence or absence of 1 mM ATP. Reactions were adjusted to a final concentration of 15 mM salt in a total volume of 20 μl, incubated for 30 min at 37°C and terminated by addition of 4 μl of loading buffer (10 mM Tris HCl pH 7.5, 1 mM EDTA, 0.5% SDS, 0.1% bromophenol blue and 20% glycerol). Samples were resolved on 12% native polyacrylamide gels in 1X TAE buffer (pH 8.3), dried and visualized on a Typhoon TRIO scanner (GE Healthcare), and quantified using Image Quant TL software (GE Healthcare).

### Electrophoretic mobility shift assay (EMSA)

To generate the heteroduplex substrates we first labelled the top strand with [γ-^32^P]dATP (Perkin Elmer) and T4 kinase (New England Biolabs). For the annealing reaction, the labelled oligonucleotide was mixed with the corresponding complementary strand and boiled for 5 min in a 1l beaker, then the water was left to cool down to <30°C. All labelled substrates were purified using illustra Microspin G50 columns (GE Healthcare).

EMSAs were performed as described in [58]. Briefly, 30 fmol of labelled DNA substrate was incubated with protein in a 20μl reaction containing 10mM Hepes-KOH (pH 7.5), 10 mM KCl, 3.3 mM MgCl2, 1 mM EDTA, 2.5 mM DTT, and 400 ng poly(dI-dC) (Sigma). For His-Rep68, 100 ng of protein was used and the reaction was set up using 15 mM NaCl. For the His-TrwC/Rep chimeric protein, the best conditions were 200 ng of protein and 75 mM NaCl. Cold competitor at 10 to 90-fold excess was added to the reactions where appropriate. After incubation for 20 min at room temperature, samples were spun down and 3 μl of loading buffer (0.25X TBE, 40% sucrose, 1% bromopheonol blue, 1% xylene cyanol) was added. The reactions were analysed on a native 6% polyacrylamide gel in 0.25X TBE. After the run, gels were treated as for the DNA helicase assay.

### Supercoiled (sc) DNA nicking assay

scDNA nicking activity for His-Rep68 was assayed as described in [59] with the following modifications. Briefly, assays were performed in 30 μl reactions containing 30 mM HEPES-KOH (pH 7.5), 7 mM MgCl_2_, 0.5 mM DTT, 1.33 mM ATP, 13.33 mM creatine phosphate (Sigma), 0.33 μg creatine phosphokinase (Sigma) and 15 mM NaCl. 100 ng scDNA plasmid and 300 ng of purified His-Rep68 were used for individual reactions. His-Rep68 reactions were incubated for 10 min at 35°C before adding stop solution. To test the His-TrwC/Rep chimera scDNA nicking activity, 600 ng of purified protein and 100 ng of scDNA plasmid were incubated in a buffer containing 30 mM HEPES-KOH (pH 7.5), 7 mM MgCl_2_, 0.5 mM DTT and 50 nM NaCl. His-TrwC/Rep reactions were incubated for 45 min at 35°C. All reactions (His-Rep68 as well as His-TrwC/Rep) were terminated by adding 10 μl of stop reaction solution (proteinase K [1.2 μg/μl], 0.5% SDS and 30 mM EDTA pH7.5) and incubated for 5 min at 35°C. Samples were resolved in a 1% agarose gel (1X TAE), which was subsequently stained with ethidium bromide (0.3 μg/ml) in 1X TAE. scDNA plasmids used in this assay were pRVK (contains AAVS1 nucleotide 1 to 3536 bp [53]) and pSU1186 (contains the full 402 bp *oriTw* (54)) (Table 1). Bands were analyzed using the gel image analysis software Image Lab 6.0.1 from Bio-Rad.

### Analytical ultracentrifugation

Sedimentation velocity experiments were carried out using a Beckman Optima XL-I analytical ultracentrifuge (Beckman Coulter Inc.) equipped with a four and four-position AN-60Ti rotor. TrwC/Rep chimeric protein samples were loaded in the cells, using the same buffer used in the final purification step. Samples in double sector cells were centrifuged at 25,000 rpm. In all experiments, temperature was kept at 20°C. A range of protein concentration from 2.5 to 10 μM was used to evaluate the potential oligomerization of the TrwC/Rep chimera. Sedimentation profiles for the protein were recorded using UV absorption (280 nm) scanning optics. The DNA used was *oriTw*(25+8) which was modified by adding a dATP at the 5’ end, which gives rise to a 34-mer oligonucleotide. For the formation of the complexes, the 34-mer oligonucleotide modified with a 5’ 6-carboxyfluorescein molecule (Integrated DNA Technologies) was incubated with the protein. The chimeric protein concentration was 3.32 μM (0.25 mg/ml), while the *oriTw*(34 mer) DNA concentration was 0.83 μM, for the protein/DNA ratio to be 4:1. Sedimentation profile analyses for the complexes were recorded using scanning optics at 492 nm. The SEDFIT program was used to analyse the results, and the Lamm equation [60] was used to calculate the sedimentation coefficient distribution profiles. The *S* theoretical values for the oligomeric states were obtained as described before [36].

### Fluorescence polarization assays

In order to determine the Kd for the DNA/protein interaction, fluorescence polarization experiments were performed on a variable temperature fluorescence polarization system (Beacon 2000). The DNA substrate used was the fluorescent-tagged DNA mentioned before. Reactions were performed in a volume of 120 μl with a buffer containing 25 mM HEPES (pH 7.0), 200 mM NaCl, and a final concentration of 5 nM DNA. Protein concentrations ranged from 0 to 750 nM. Samples were incubated at 25°C for 30 min prior to measurement. Binding activity was analyzed at an excitation of 492 nm and an emission of 520 nm [61]. Data analysis was performed as described by Yoon-Robarts *et al*. [57].

### Site-specific integration assay in bacteria

Site-specific integration of a suicide plasmid into an *oriT*-containing plasmid in recipient bacteria was assayed as previously described [43]. Matings were done using the S17.1 λpir strain [62] as donor bacteria containing pCMS11 (Table 1), the suicide plasmid harbouring RP4 *oriT* (*oriT*_*P*_), R388 *oriT* (*oriT*_*W*_) and chloramphenicol resistance gene, which only replicates in Pir strains. RP4_TraI mobilizes the suicide plasmid from the donor to DH5α [47] recipient bacteria containing a plasmid with an *oriT*_*W*_. *trwC*, the relaxase domain of *trwC* (*N293-trwC*), or *trwC/rep* chimera were expressed in the recipient under the control of the T7 promoter, to avoid an overexpression-induced toxic effect in the cell, as previously described [43]. Integrants were selected in plates containing nalidixic acid (Nx; 20 μg/ml) and chloramphenicol (Cm; 25 μg/ml). Only bacteria harboring a cointegrate between the suicide plasmid and the recipient *oriT*-containing plasmid can survive, since the suicide plasmid cannot be replicated in the *pir—*recipient bacteria. Conjugative transfer of the suicide plasmid was measured in parallel using DH5α λpir [63] as a recipient. Transfer efficiency was always close to 100% transconjugates/donor. Putative integrants (Nx^R^ Cm^R^ colonies) were analysed as follows. DNA was extracted by Instagene (Bio-Rad) and used for PCR analysis. Primers P1 (5’- AGCGGATAACAATTTCACACAGGA-3’) and P2 (5’-GCAGGATCCGCTAAFCTTTGTCGGTCATTTCGA-3’) (43) annealing to the recipient plasmid and the donor plasmid downstream *oriT*_*W*,_ respectively, were used to amplify a 1.2 kb amplicon, expected only for the cointegrate molecules. PCR products were extracted and the DNA sequence was determined using the primer P1.

### Site-specific integration assay in human cells

The C17 cell line, a derivative of the 293 cells line [64] which constitutively expresses EBNA-1, was used to facilitate propagation of the p220.2 extrachromosomal nuclear episomes. Cells were grown in DMEM plus 10% FBS, with G418 (Thermo Fisher) at 1200 μg/ml (to maintain EBNA-1 gene). 1 μg of EBV-based episome containing the integration site (p220.2::*AAVS1*(kb0-0.51), or episomes pLA58 (containg *oriTw*) or pLA59 (containing *oriTw* in the opposite direction), were transfected into C17 cells with 6 μl of Lipofectamine 2000. After 30h of transfection, the cells were split 1:10 into media containing G418 at 1200 μg/ml and Hygromycin B (Thermo Fisher) at 200 μg/ml (to select for the episomes). Cells at very low passage (2–4) were used to assure high number of episomes per cell. EBV plasmid-containing C17 cells containing the indicated episome were grown to confluency in DMEM+10%FBS, G418 (1200 μg/ml) and Hygromycin B (200 μg/ml) in T225 flasks. Then, 4.5x10^6^ cells were split into 100-mm plates. Ten plates were used per condition and were grown without antibiotics for 48 h, until they reached 60–80% confluency.

To perform the integration assay for the chimeric protein or TrwC, the plasmid to be integrated, i.e. the suicide plasmid pCMS11, and either pLA117 (expressing *trwC/rep* chimera) or pLA119 (expressing *trwC*) were transfected in equimolar amounts (10 μg total DNA) by calcium phosphate coprecipitation [65]. As a negative control, pCMS11 (4.5 μg) was cotransfected with salmon sperm DNA up to 10 μg. At 24 h after transfection, DNaseI 50 units/ml (Roche) was added to the cells. Transfection efficiency was checked by flow citometry using pIRES-eGFP plasmid. For Rep68-mediated integration, cells were infected with wild type AAV2, which encodes Rep68 and contains the target DNA sequence for integration into the episome. Viral particles purified from an iodixanol gradient [66] were added at an MOI of 10^6^ to assure 100% transduction efficiency and increase the chance of episomal integration.

72 h after transfection/infection, cells were harvested to isolate the episomes using a modified Hirt extraction procedure [67]. Cells were first washed once with PBS 1X. Then, 1 ml of lysis solution (SDS 0.6%, EDTA 10 mM, Tris pH 7.4 10 mM) was added per 100-mm plate and left for 10 min at room temperature. Cells were then collected and transferred to a tube and were subjected to digestion with proteinase K (Sigma) at a final concentration of 50 μg/ml for 2 h at 37°C. NaCl 1M final concentration was used to precipitate the high molecular weight DNA. Tubes were incubated overnight on ice. The next day, tubes were centrifuged at 48000 *g* and supernatants were transferred to a new tube to proceed with a phenol-chloroform-isoamyl alcohol extraction. Upper phase was transferred to a new tube and sodium acetate at 0.3M final concentration and 0.7 vol. of isopropanol were added and the tubes were kept at -20°C overnight. Pellets were obtained by centrifugation, rinsed with 70% ethanol and dried. The extrachromosomal DNA was resuspended in 200 μl of 10 mM Tris HCl, 1 mM EDTA pH 8.

2 μl of the episomes were electroporated into SURE bacteria (Stratagene). The electroporated bacteria (30 μl) were diluted in 1 ml of LB and incubated at 30°C for 1 h. 100 μl of the electroporated bacteria were plated on LB/Amp plates to determine the number of Amp^R^ colonies. The remaining fraction was adjusted to produce 200–300 colonies per plate, which were duplicated on nylon membranes (Roche). Imprints made from the plates were incubated on LB/Amp plates until colonies were 0.5-1mm in diameter. Colony hybridization of the replica filters was carried out with a radioactive probe for TrwC/ TrwC-Rep chimera experiments prepared from PCR on pCMS11 with primers 5’-TTTTCCGCTGCATAACCCTGCTT-3’ and 5’-ATGATTGAACAAGATGGATTGCAC-3’, avoiding amplification of the *oriTw*. For the Rep68 experiment a *cap* probe was used. Hybridization was performed in nylon wash buffer at 65°C for 20 h [68]. For TrwC and TrwC-Rep experiments, bacteria were also plated in LB Amp/Cm to detect cointegrate molecules [episomes that are resistant to ampicillin (Ap^R^) and chloramphenicol (Cm^R^), derived from pCMS11].

### Bioinformatics tools

Ribbon diagrams of the N-terminal part of TrwC (PDB ID: 1OSB) and Rep (PDB ID: 1M55), as well as the alignment of the two proteins, were generated using PyMOL Molecular Graphics System (Version 1.6 Schrödinger, LLC).

## Results

### Design and validation of the bacterial-viral chimeric protein TrwC/Rep

In order to test the functional conservation of HUH endonucleases, we engineered a chimera between two distantly related HUH superfamily members: the Rep68 protein from the human virus AAV2 and the conjugative relaxase TrwC from the bacterial plasmid R388.

**Fig 1:**
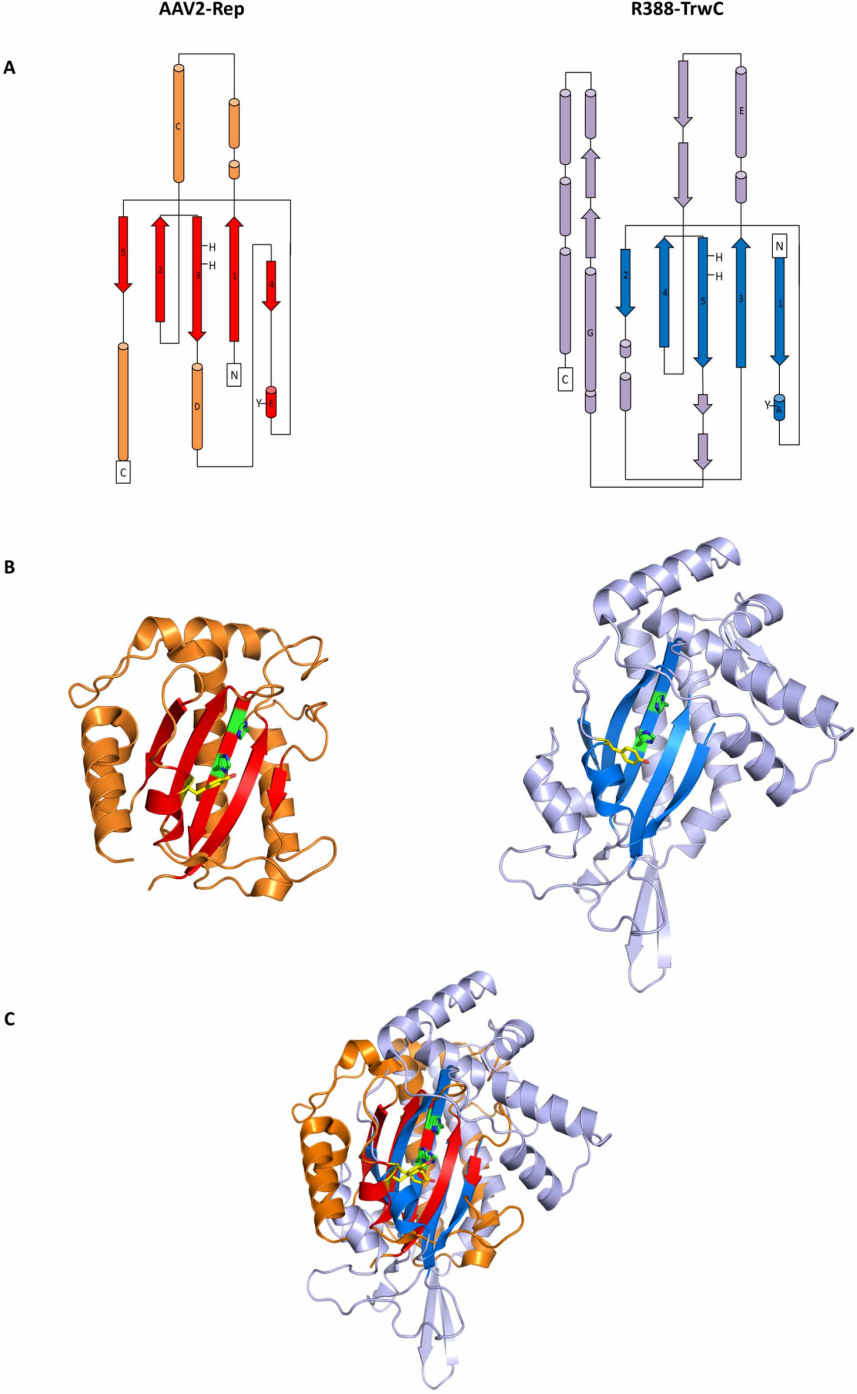
Structural similarities between the N-terminal domains of AAV2-Rep and R388-TrwC. **(A)** Topology diagram of the OBD domain of Rep (left) and relaxase domain of TrwC (right). The portion of the structure that forms the core active site typical of HUH endonucleases, consisting of five anti-parallel β-sheets and one α-helix and containing the active site tyrosine (helix E in AAV-Rep and helix A in R388-TrwC), is highlighted in red and blue, respectively. Helices with some degree of overlap are shown with their name (C and D in AAV-Rep, E and G in R388-TrwC). **(B)** Ribbon diagrams shown in the same orientation as in (A), with the active site tyrosine highlighted in yellow and the consensus HUH motif in green. TrwC has 2 active tyrosines, Tyr18 and Tyr26, but only Tyr18 is mapped in the TrwC structure. **(C)** Structural alignment of the HUH domains of Rep (red) and TrwC (blue) showing a high degree of similarity between the active sites of the two proteins, while no similarities are noted in the rest of the protein structure (shown in orange and violet, respectively).

Fig 1 shows a comparison of the 3D structure of the N-terminal domains of the two proteins [38, 69], which harbor the catalytic HUH and Tyr(s) motifs and display the catalytic endonuclease activity, as detailed in the Introduction. In Rep68, the N-terminal OBD domain is formed by 5 central antiparallel β-strands, 6 α-helices, and 7 loop regions [70] (Fig 1A and 1B, left panels). The endonuclease domain of TrwC consists of the 5 central antiparallel β-strands conserved among HUH proteins plus 6 additional β-strands, 11 α-helices and 7 turns (38) (Fig 1A and 1B, right panels).

Superimposition of Rep68 and TrwC (Fig 1C) shows that the five-stranded antiparallel β-sheets containing the HUH core, align with an RMSD of 2.1 Å. The Y motifs containing the catalytic Tyr are located at α-helices occupying similar positions in the active site, but are topologically at different positions with respect to the HUH motif due to a circular permutation of the Y motifs [15, 71]. Aside from the HUH motif similarity, some degree of overlap is also observed for the two long α-helices behind the central β-sheets (helices C and D in AAV-Rep68, E and G in R388-TrwC), even though they are connected differently to the active site core. The rest of the structure however, differs strongly between Rep68 and TrwC. R388-TrwC has additional α -helices, β -sheets and long loops that stem further away from the active site (Fig 1C).

**Fig 2:**
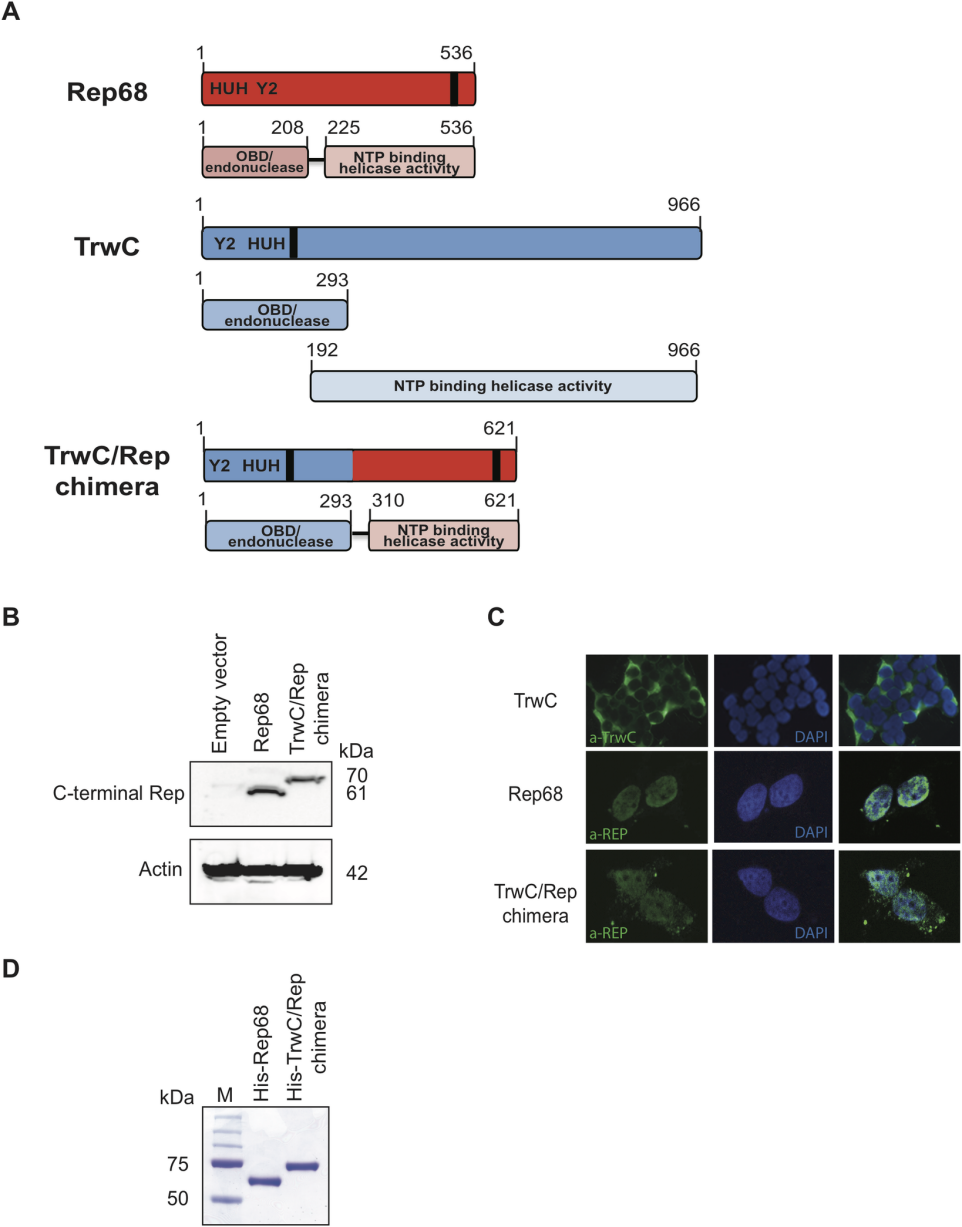
Design and validation of the TrwC/Rep chimeric protein. **(A)** Schematic representation of Rep68, TrwC and the TrwC/Rep chimera. For each protein, overall configuration and functional domains are shown. The position of the HUH and Y2 motif, and the NLS (black bar) are indicated. Amino acid (aa) positions are indicated above each protein. **(B)** Western blot analysis of Rep68 and TrwC/Rep chimera expressed in 293T cells. Molecular weight in kDa is shown on the right. Antibodies used to detect proteins are shown on the left. **(C)** Localization of TrwC, Rep68 and TrwC/Rep chimera in 293T cells visualized by immunofluorescence microscopy. Images are shown at 60X magnification. **(D)** Assessment of the purity of His-Rep68 and His-TrwC/Rep chimera purified by nickel affinity and gel filtration chromatography. 200 ng of protein was loaded onto a 12% SDS-PAGE gel and subsequently stained with Coomassie Brilliant blue to visualize the quality of the purified protein. Marker (M) is shown on the left; molecular weight from top to bottom is: 250, 150, 100, 75, 50 kDa.

We generated a TrwC-Rep chimera by fusing the relaxase domain of TrwC (N293-TrwC), capable of sequence-specific DNA nicking and strand-transfer reactions [38, 41], to the Rep68 C-terminal domain, responsible for NTP-binding and DNA helicase activity. The Rep68 C-terminal domain spans residues 225 to 536, which harbors the DNA helicase activity [20, 72, 73], and amino acids 209–224, which are required for oligomerization of the Rep78/68 proteins, and thus protein functionality [34, 35]. Fig 2A shows the functional domains in Rep68, TrwC, and the chimeric TrwC/Rep protein.

To test if the construct is stable in human cells, we cloned the *trwC/rep* chimeric gene into a eukaryotic expression vector. Transfection of 293T cells with this vector showed that the chimeric protein exhibits the expected molecular weight and is stably expressed in human cells (Fig 2B). Next, we determined the cellular localization of the proteins in cells transfected with the TrwC/Rep, TrwC, or Rep68 expression vectors. TrwC has a nuclear localization signal (NLS) in the N-terminal domain and immunofluorescence studies have shown that TrwC is located both in the nucleus and the cytoplasm, with the latter being the predominant cellular localization (52). Rep68 on the other hand contains a NLS in its C-terminal domain, which drives the protein to the nucleus [74]. Not surprisingly, the TrwC/Rep protein mainly showed nuclear localization, although some level of cytoplasmic distribution could be observed (Fig 2C).

### The TrwC/Rep chimera retains the enzymatic activities of both parental proteins

**Fig 3:**
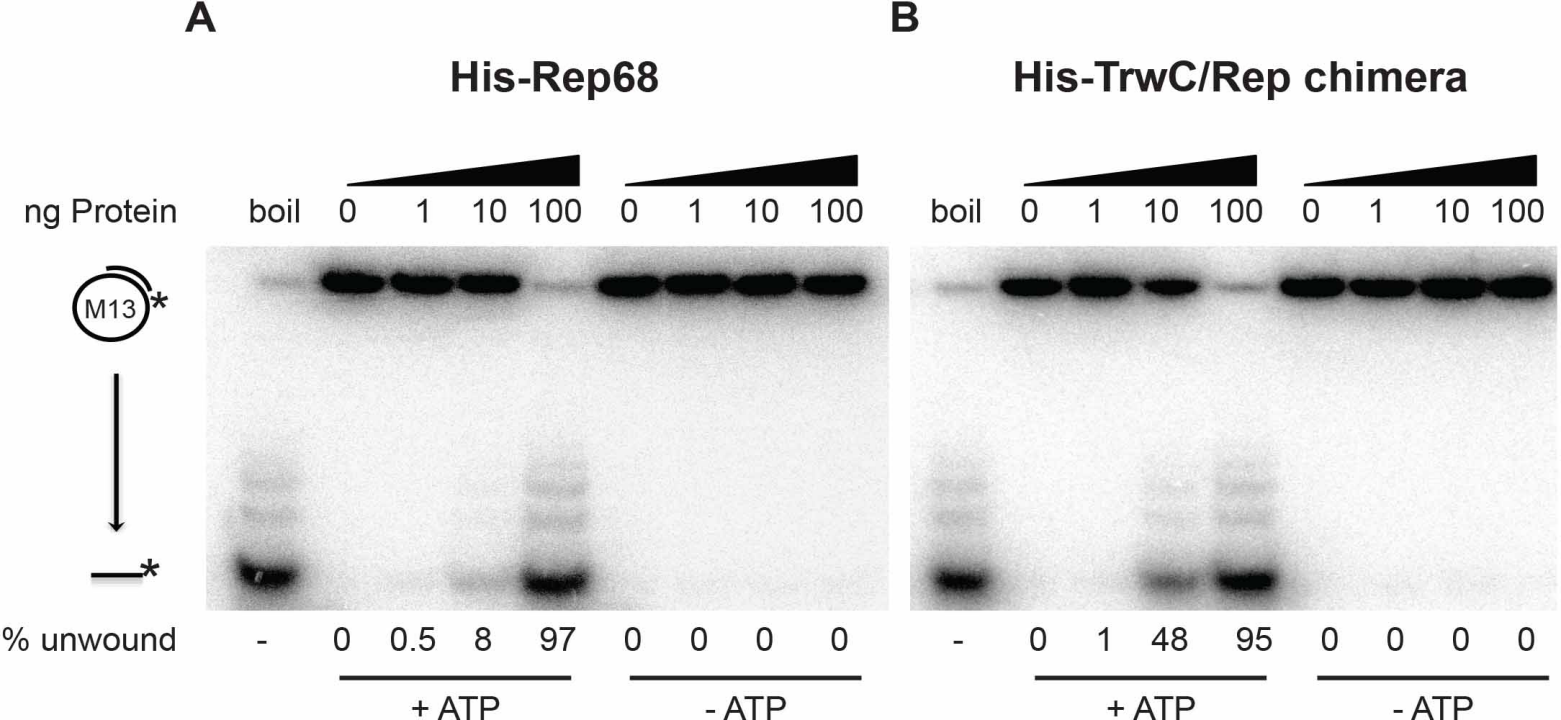
DNA helicase activity of Rep68 and TrwC/Rep. Increasing amounts of Rep68 and TrwC/Rep chimeric protein (0, 1, 10, 100 ng of protein) were assayed in the presence or absence of ATP. “boil” indicates heat-denatured substrate, used as a positive control. Products of the reaction were resolved on 12% native polyacrylamide gels. The diagram to the left of the gel shows the position of the substrate (partial duplex M13 DNA) and unwound labelled ssDNA. The percent of unwound substrate was quantified using the Image Quant TL software. The helicase efficiency percentage is a representative example obtained from 3 independent experiments (n = 3).

In order to characterize the protein biochemically, the *trwC/rep* chimeric gene was cloned into a pET15b vector to add an N-terminal His-tag for simple purification. The resulting plasmid pLA106 was expressed in *E*.*coli* and the His-TrwC/Rep was purified as described in the Materials and Methods section; a His-Rep68 protein was purified in parallel. Both proteins were run on a SDS-PAGE gel to confirm final purity of the preparations (Fig 2D). To assess whether the chimera was functional, we tested the various enzymatic activities displayed by the parental proteins. DNA helicase activity was verified using an M13-derived partially duplexed DNA substrate, as used previously to assess the helicase activity of Rep40 (minimal helicase domain, amino acids 225–536) [57]. A DNA helicase assay using increasing amounts of proteins showed that the chimeric protein is equally active in unwinding DNA as the Rep68 protein when 100 ng of protein is used (Fig 3). Surprisingly, the chimeric protein also displayed robust helicase activity at 10 ng, conditions under which Rep68 unwinds only 8% of the radiolabeled substrate. To confirm that the observed DNA unwinding is indeed a product of DNA helicase activity, we tested the dependence on ATP. As expected, neither the chimeric protein, nor Rep68 were able to unwind DNA in the absence of ATP, confirming that the ssDNA obtained is the result of specific helicase action (Fig 3). These results indicate that the DNA helicase domain in the TrwC/Rep chimera is folded correctly and fully supports dsDNA unwinding.

**Fig 4:**
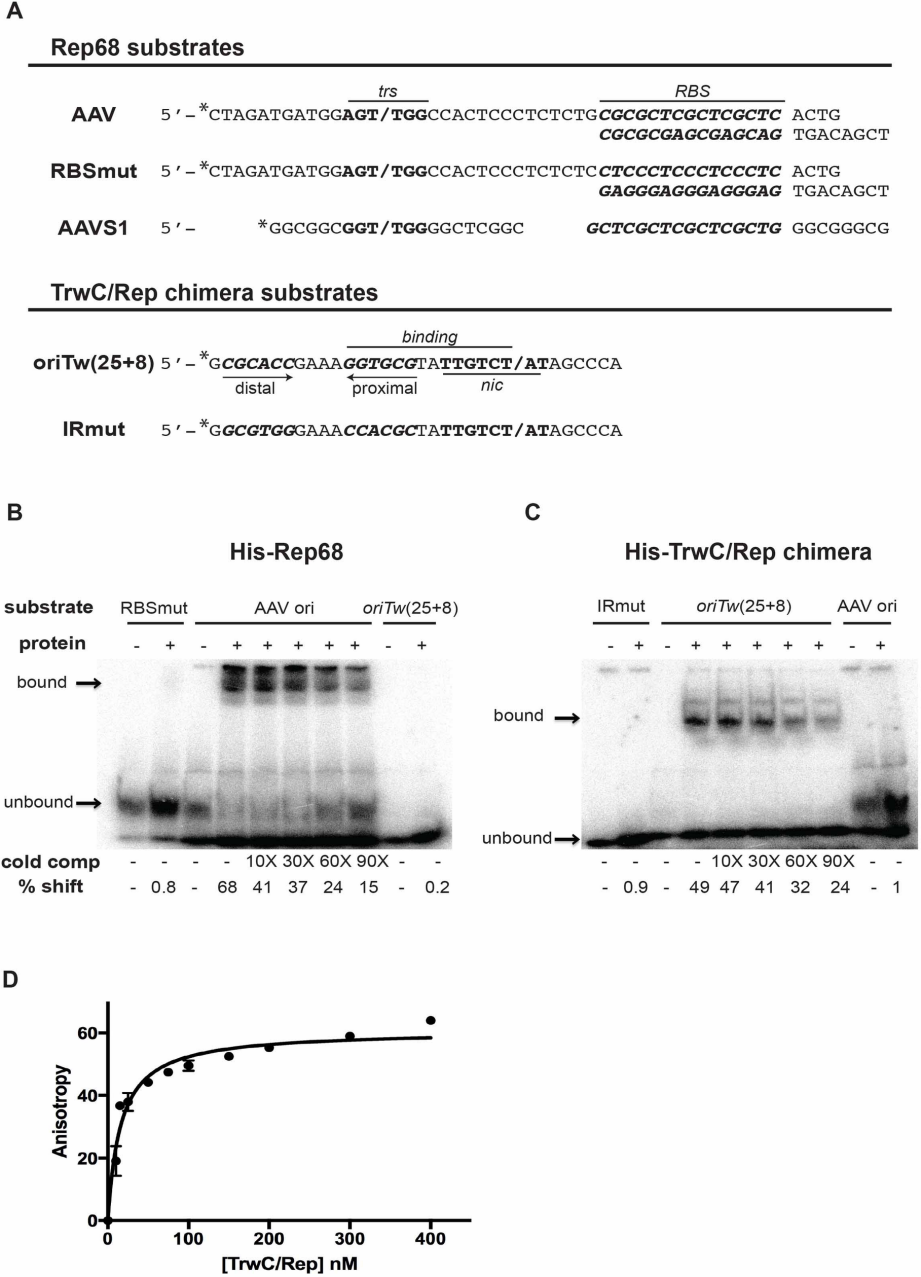
DNA binding activity of Rep68 and TrwC/Rep. **(A)** Rep68 and TrwC/Rep DNA substrates used for binding assays. Rep68 substrates were an AAV ori heteroduplex, and an equivalent AAV ori substrate mutated in the RBS sequence (RBSmut). *trs* is shown in boldface; nicking site is indicated by a slash. RBS is highlighted in bold italic. The *AAVS1* minimal sequence (30) is presented below. TrwC/Rep chimera substrates were oligonucleotides *oriTw*(25+8) and *oriTw*(25+8) mutated in the inverted repeat IR (IRmut). Horizontal lines and nucleotides highlighted in boldface show sequence requirements for binding and nicking activities [77]. The *nic* site is represented by a slash. The IR recognized during binding is indicated with arrows; distal and proximal arms are shown. * represents the radioactively labelled strand. **(B)** and **(C)** EMSA assays with His-Rep68 and His-TrwC/Rep chimera, respectively. 30 fmol of the indicated radiolabelled substrates were incubated either with 100 ng of His-Rep68 or 200 ng of His-TrwC/Rep. All reactions contain 400 ng of poly(dI-dC) as nonspecific DNA. Competition assays were done using cold competitor DNA at 10- to 90-fold molar excess. Products were analysed on a native 6% polyacrylamide gel. Percentage of bound substrate (“% shift”) was calculated using Image Quant TL software. Bound and unbound products of the reaction are indicated with arrows. The DNA binding percentage is a representative example obtained from 3 independent experiments (n = 3). **(D)** Fluorescence polarization assay with *oriTw*(25+8) DNA labelled with carboxyfluorescein at 5 mM concentration. Binding was performed in 25 mM HEPES (pH 7.0), 200 mM NaCl at room temperature. Data analysis was performed as described by Yoon-Robarts *et al*. [57].

Both Rep68 and TrwC have the ability to specifically bind their cognate DNA substrate *in vitro*. Rep68 binds dsDNA at the RBS sequence within the hairpin shaped ITR [72, 75, 76], whereas N293-TrwC binds to the proximal arm of the inverted repeat IR_2_ at *oriT*_*w*_ [41, 77]. To assess DNA binding *in vitro*, we used heteroduplex substrates encompassing the AAV ori region, containing the *trs* and RBS sequences for Rep68, and ssDNA containing the TrwC binding and *nic* sites (Fig 4A). We first optimized the binding conditions for each protein by incubating them with their corresponding substrates at different salt concentrations. We found that the optimal salt concentration for Rep68 was 15 mM NaCl, whereas the TrwC/Rep chimera binds most efficiently at 75 mM (data not shown). We then assessed if the chimeric protein specifically targeted the TrwC binding site by performing EMSA assays using the aforementioned DNA structures; substrates carrying mutations in the binding sites were included as controls [22]. As expected, the parental protein, Rep68, induced a shift of approximately 70% of the radiolabeled AAV ori substrate, but did not bind to an RBS-mutated substrate (RBSmut) or the TrwC substrate (*oriT*_*w*_(25+8)) (Fig 4B). The TrwC/Rep chimera was found to bind to the *oriT*_*w*_(25+8) oligonucleotide, resulting in a mobility shift of 49% of the radiolabeled DNA (Fig 4C). The shift decreased to 24% when binding was competed by a 90-fold excess of cold *oriT*_*w*_(25+8) competitor. The interaction was highly substrate-specific, as the TrwC/Rep chimera did not bind to a mutated binding site (IRmut) or the Rep68 substrate (AAV ori) (Fig 4C). These results show that the chimeric protein is able to recognize and bind to the TrwC substrate specifically.

To determine the affinity of binding of the chimeric protein, we carried out polarization assays by fluorescence anisotropy using the 34-mer oligonucleotide *oriTw*. The association constant (Kd) for TrwC/Rep chimera was 16 nM, which indicates a high affinity binding of the protein to its substrate (Fig 4D). The dissociation constant for N293-TrwC with the same *oriTw* oligonucleotide was previously determined as 30 nM [77], indicating that both proteins present high affinity to the substrate.

**Fig 5:**
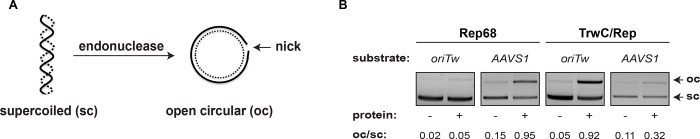
Rep68 and TrwC/Rep nick sc plasmid DNA containing binding and nicking sites. **(A)** Schematic representation of the scDNA nicking assay. When incubating a sc plasmid with an endonuclease, the protein nicks specifically inducing relaxation of the plasmid (open circular, oc), which will result in slower migrating DNA species when visualised on an agarose gel. **(B)** His-Rep68 and His-TrwC/Rep-mediated scDNA nicking. 100 ng of the plasmid containing the target sequence indicated on top of the gels was incubated as described in Materials and Methods with either purified His-Rep68 (left panels) or His-TrwC/Rep chimera (right panels). Products were resolved on 1% agarose gels, subsequently stained with ethidium bromide. “-“: sc plasmid incubated in the reaction buffer and processed as described above, but in the absence of protein. sc and oc products are indicated with arrows. The oc/sc ratios of quantified DNA bands are indicated at the bottom of the panels. The presented gels are representative of 3 independent experiments (n = 3).

To address whether the TrwC/Rep chimera maintained the ability to bind, melt, and nick the target scDNA, we performed a scDNA nicking experiment. To this end, 100 ng of scDNA containing either full-length *oriTw* (402 bp, pSU1186), or *AAVS1* (3525 bp, pRVK) were incubated as described in Materials and Methods. If endonuclease activity is present, the plasmid conformation will change from sc to an open circular (oc) form (Fig 5A), which is easily visualized on an agarose gel. As shown in Fig 5B, the His-TrwC/Rep chimera showed nicking activity on the *oriTw* containing plasmid, while it did not cleave the *AAVS1* containing plasmid, indicating that the observed nicking activity requires recognition of a specific cleavage site. In comparison, incubation with His-Rep68 did not alter the *oriT*-containing plasmid, as expected, while it showed the previously observed pattern when acting on *AAVS1* [28, 35]. These results demonstrate that the TrwC/Rep protein engages with the TrwC scDNA substrate, and is able to perform the subsequent melting and nicking reactions.

### The TrwC/Rep chimera oligomerizes in the presence of DNA

**Fig 6:**
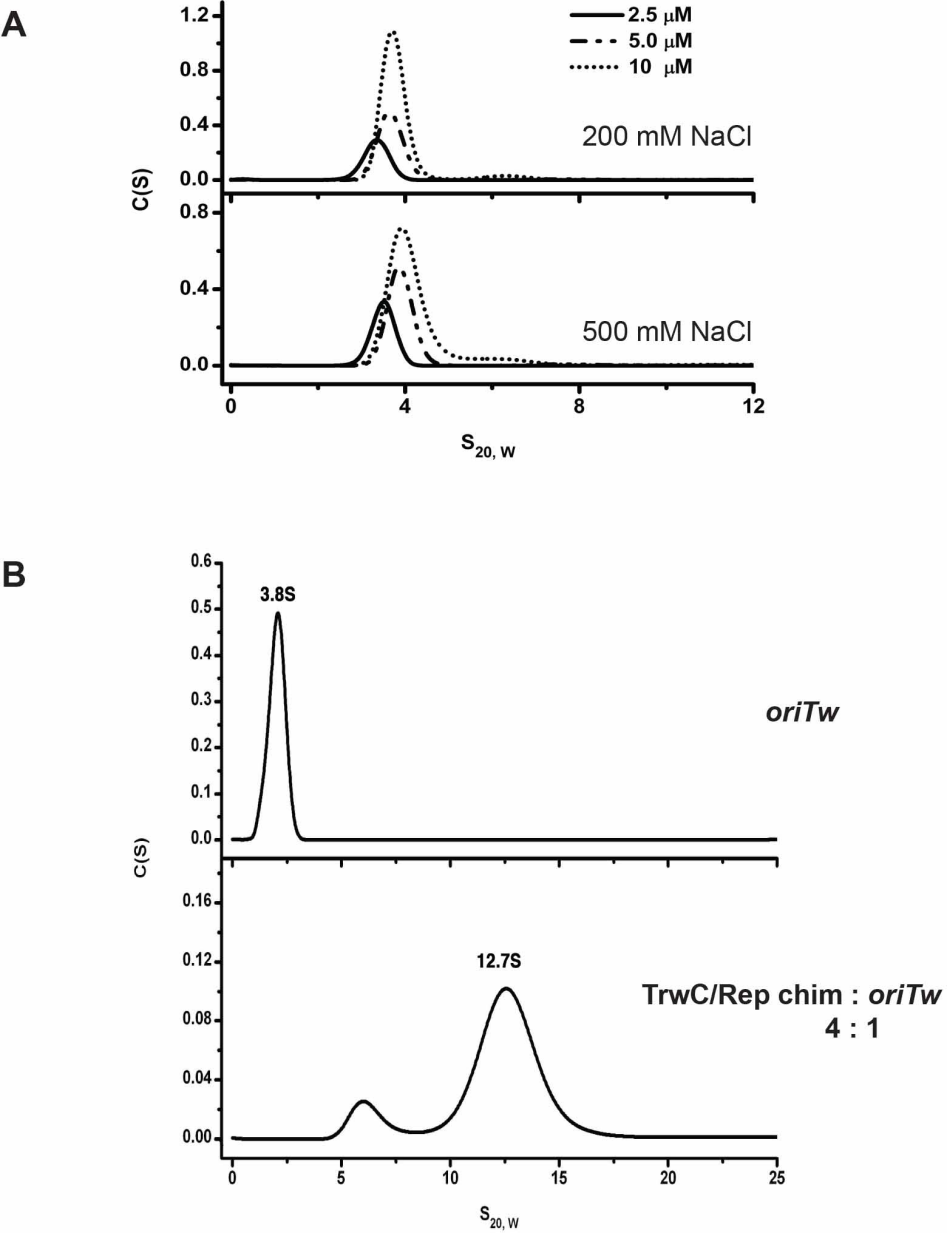
Sedimentation velocity analysis of the TrwC/Rep chimera. **(A)** Sedimentation profiles of the TrwC/Rep chimera at three different concentrations of protein– 2.5, 5 and 10 μM—in 200 mM NaCl (top) or 500 mM NaCl (bottom), obtained using the SEDFIT programme. **(B)** Sedimentation profile of TrwC/Rep incubated with its specific substrate *oriTw*(25+8) oligonucleotide. Top: in the absence of DNA, the substrate sediments with a sedimentation coefficient of 3.8S; Bottom: at a 4:1 protein:DNA ratio, the analysis shows a peak of 12.7S, suggestive of hexamer species based on the theoretical S_20,w_ values obtained as described before [36].

The TrwC/Rep chimera was designed to contain the relaxase domain of TrwC, previously shown to behave as a monomer irrespectively of the presence of its target DNA [77], and the complete C-terminal region of Rep68, including the interdomain linker (residues 209–536) that connects the OBD and the DNA helicase domain, shown to be essential for Rep68 oligomerization into 7-mer and 8-mer rings [34]. To investigate the oligomeric behavior of the TrwC/Rep chimera, we subjected the protein to sedimentation velocity experiments using concentrations of 2.5, 5 and 10 μM (0.2, 0.4, and 0.8 mg/ml respectively). The sedimentation coefficient values ranged from ~3.8S to 4S, which corresponds to a monomer (Fig 6A, top). A higher salt concentration of 500 mM did not show any higher-order oligomers (Fig 6A, bottom). We next proceeded to study how the chimeric protein behaves in the presence of its specific target DNA. Therefore, the protein was incubated with fluorescently labelled *oriTw* (34mer) substrate and subjected to analytical ultracentrifugation (Fig 6B). Using a 4:1 protein/DNA ratio, the sedimentation profile shows a major species sedimenting at 12.7S (Fig 6B, bottom). Assuming a globular-shaped complex, the calculated sedimentation coefficient for a hexamer is approximately 12.7S. Further studies would be required for an accurate stoichiometry determination. Nevertheless, these results suggest that in the presence of DNA, the TrwC/Rep chimera forms a high order oligomeric complex of at least six subunits.

### The TrwC/Rep chimera catalyses site-specific integration

**Fig 7:**
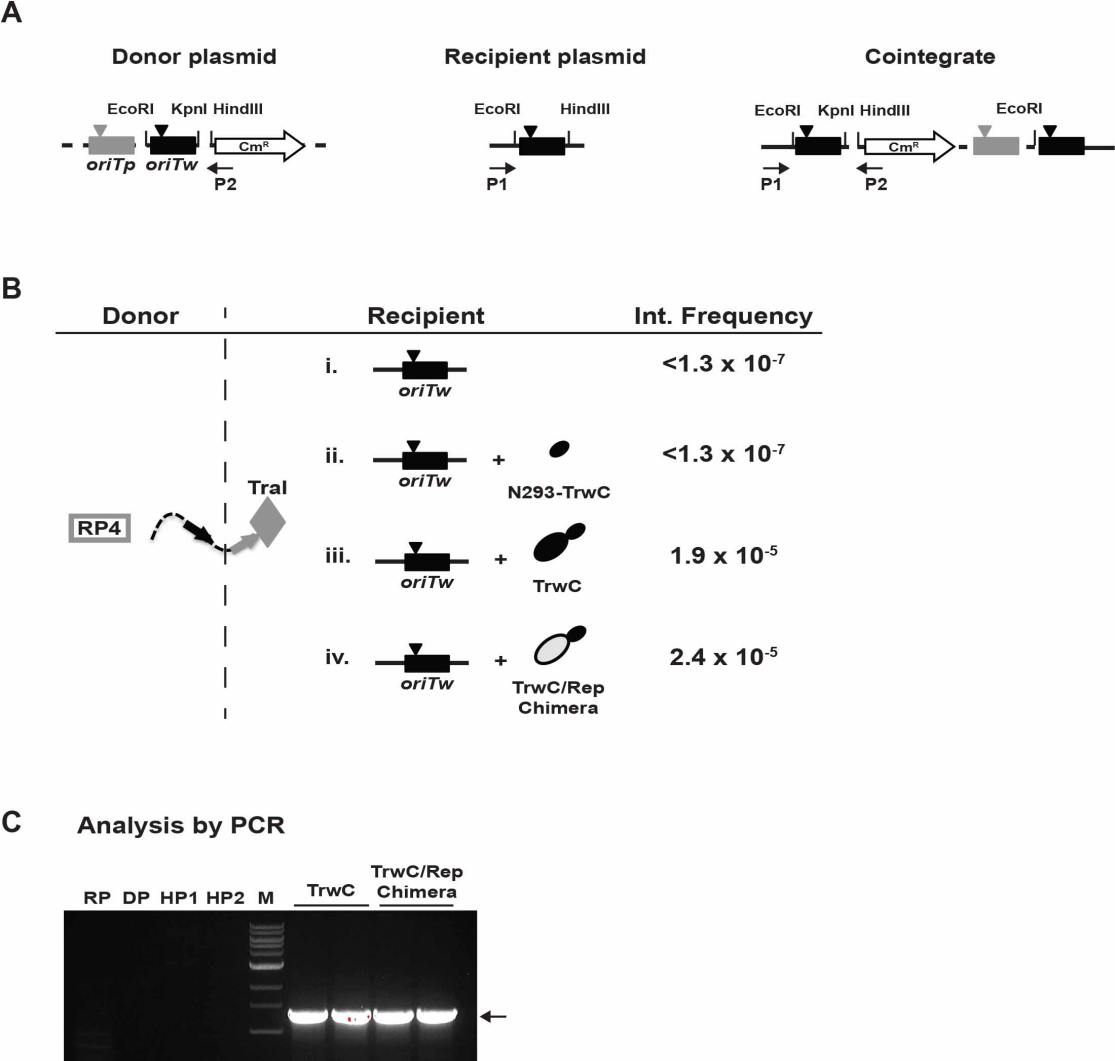
Site-specific integration assay. **(A)** Scheme of the plasmids used in the assay and the cointegrate molecule obtained. The donor suicide plasmid pCMS11 (pR6K::*oriTp oriTw*) is represented with a dotted line (left panel). *oriTp* and *oriTw*, the origins of transfer recognized by the relaxases of plasmids RP4 and R388 respectively, are shown as boxes in grey and black, respectively. *nic* sites are indicated with triangles in the respective colour code. The chloramphenicol resistance gene is represented as a white arrow (CmR). Recipient plasmid (pLA32) containing *oriTw* is shown as a black line (center panel). The last plasmid is the result of the integration reaction and is referred to as the cointegrate molecule (right panel). Relevant restriction sites are indicated in each plasmid. P1 and P2 are the oligonucleotides used to amplify the DNA of the integrants. **(B)** Representation of the integration assay in which the integrase is expressed in the recipient bacteria. The suicide plasmid is mobilized by the TraI relaxase (grey diamond) from the RP4 conjugative system (represented by a square) in a S17.1λpir donor strain to a DH5α recipient harbouring the *oriTw*-containing plasmid. Four different assays are represented: (i) no expression of integrases in the recipient bacteria (negative control); (ii-iv) recipient bacteria contain a plasmid coding for the relaxase domain of TrwC (N293-TrwC), full-length TrwC, or the TrwC/Rep chimera, respectively. TrwC protein is represented as black ellipses, and the Rep domain, in light gray. Integration frequencies obtained are also shown in the figure. Data are the mean of five independent experiments. Frequency is given as integrants/donors. **(C)** PCR analysis using primers P1 and P2. Agarose gel showing PCR amplicons (1.2 kb) obtained as a result of the integration reaction mediated by TrwC or TrwC/Rep chimera. RP, recipient plasmid; DP, donor plasmid; HP1, helper plasmid coding for TrwC protein; HP2, helper plasmid for TrwC/Rep chimera expression; M (1kb ladder): 10-8-6-5-4-3-2-1.5–1 kb.

Unexpectedly for a conjugative relaxase, TrwC is able to integrate the transferred ssDNA site-specifically into both an *oriTw*-containing plasmid and the bacterial chromosome [17, 43]. To determine if the TrwC/Rep chimera is able to coordinate the different biochemical activities required for achieving an integration reaction, we performed an integration assay in bacteria. To this end, we used an assay in which the integrase is expressed in the recipient bacteria [43], thereby circumventing the requirement for a relaxase that can mediate conjugative DNA transfer. The integration assay and plasmids used are depicted in Fig 7A and 7B. In this assay, a donor suicide plasmid (pR6K::*oriTp oriTw*) was mobilized into the recipient bacteria by TraI, the conjugative relaxase of the RP4 conjugative system, which recognizes *oriTp*. The recipient bacteria harbour an *oriTw*-containing plasmid (pLA32) and another plasmid expressing either N293-*trwC* (pSU1600), *trwC* (pSU1621) or *trwC/rep* chimera (pLA131).

In the presence of the TrwC/Rep chimera in the recipient cells, we observed an integration frequency of 2.4x10^-5^ (Fig 7B (iv)), which is very similar to the integration frequency obtained when TrwC supports this reaction (1.9x10^-5^ integrants/donor cell) (Fig 7B (iii)). The presence of cointegrates in recipient bacteria was confirmed by a PCR reaction using primers P1 and P2 (Fig 7A). Fig 7C shows the amplification of a 1.2 kb amplicon, for which sequence analysis confirmed the presence of the expected region of the cointegrate molecule (data not shown). No integrants were obtained in the presence of N293-TrwC (Fig 7B (ii)), implying that the relaxase domain alone is not sufficient to perform SSI, as reported previously [43].

This positive result prompted us to assess the potential of the chimera to perform site-specific integration in human cells, using an episomal assay similar to the one developed to determine the requirements for AAV Rep-mediated site-specific integration into human host DNA [49]. Briefly, the assay measures the formation of cointegrates between two replicons containing the target DNA. C17 cells carrying episomes with the appropriate target DNA were either infected (Rep68 control experiment) or transfected (TrwC, TrwC-Rep experiments) with target-containing DNA and the gene expressing the corresponding protein. Episomes were extracted and introduced in bacteria, where cointegrates were detected by colony hybridization with a probe against the integrating DNA.

To test TrwC and TrwC-Rep chimera for their ability to mediate site-specific integration, C17 cells carrying either pLA58 or pLA59 (p220.2 containing *oriTw* in both orientations) were transfected with the suicide plasmid pCMS11, and either pLA117 (expressing *trwC/rep* chimera) or pLA119 (expressing *trwC*). As a control for transfection efficiency, C17 cells carrying these episomes were transfected with plasmid pIRES-eGFP, and an efficiency of around 15% transfected cells was obtained (not shown). As a positive control, C17 cells carrying p220.2::*AAVS1*(kb 0–0.5) (the minimal recombination signals for AAV integration) were infected with wt AAV at a high MOI to ensure 100% infection efficiency.

The results are shown in Table 2. We reproduced the results for AAV-Rep mediated integration as described in Giraud *et al* (0.89% integration efficiency) [49]. However, when testing TrwC and TrwC-Rep, not a single colony out of approximately 14,000 and 37,000 Ap^R^ colonies screened respectively, reacted with the pCMS11 probe (Table 2). We also plated bacteria to select for the cointegrate molecules (episome+pCMS11) on plates containing ampicillin and chloramphenicol, but no colonies were detected.

**Table 2:** Site-specific integration mediated by Rep, TrwC and TrwC-Rep using and episomal assay.

Protein tested	Episome + integrating DNA	Integration	
		Hybridized colonies, no/total	%
AAV-Rep	p220.2 +AAV	0/564	<0.17
	p220.2::*AAVS1* (kb 0–0.5) +AAV	3/335	0.89
TrwC	pLA58 (*oriTw* nick antisense) + pCMS11	0/4137	<0.02
	pLA59 (*oriTw* nick sense) + pCMS11	0/10,049	<9.9x10^-3^
TrwC-Rep chimera	p220.2	0/3511	<0.02
	pLA58 (*oriTw* nick antisense)	0/3036	<0.03
	pLA58 (*oriTw* nick antisense) + pCMS11	0/15,835	<6.3x10^-3^
	pLA59 (*oriTw* nick sense)	0/6388	<0.01
	pLA59 (*oriTw* nick sense) + pCMS11	0/21,246	<4.7x10^-3^

Integration efficiency (%) is the percentage of ApR colonies which hybridized with the specific DNA probe. See Materials and Methods for details.

## Discussion

HUH endonucleases define a superfamily of proteins present across all biological kingdoms; it comprises proteins from bacterial plasmids, plant and mammalian viruses, and transposases from bacteria, archaea and eukaryotes. Their functions vary, ranging from bacterial conjugation to DNA transposition and viral replication by RCR [1]. To achieve this functional diversity, HUH proteins have evolved to combine a conserved HUH domain, which confers shared biochemical activities, with different functions, provided either in another domain or recruited from the host. Some members of this superfamily of proteins are able to perform SSI in addition to their biological role, suggesting a potential for biotechnological applications for these proteins [19]. It is not clear to date whether the ability of some of these proteins to mediate SSI is due to intrinsic catalytic differences, or to their respective interaction with host factors.

In this work, we focus on two of these members, the viral master replication protein AAV2-Rep68 and the bacterial conjugative relaxase R388-TrwC, both known to perform integration of ssDNA into a sequence-specific dsDNA target in human and bacterial cells, respectively. Both proteins have been well-characterized biochemically, rendering them suitable candidates to determine the requirements for their SSI activity through a domain swapping approach. To this end, we designed a TrwC/Rep chimeric protein composed of the N-terminal domain of TrwC, and the C-terminal domain of Rep68. The chimera contains the N293 relaxase domain of TrwC, which is able to perform sequence-specific binding and nicking on scDNA, and thus we expected the chimera to retain the HUH activities of TrwC. The chimera further contains the C-terminal SF3 helicase domain of Rep68. The two domains are joined by the interdomain linker of Rep68 that was shown to be required for Rep68 oligomerization. It is important to note that N293-TrwC is not sufficient to catalyse SSI in bacteria on its own. We wanted to test if the C-terminal domain of Rep68 could contribute to the function of the chimeric protein by allowing it to interact with DNA structures and proteins from the host cell that may be required to achieve SSI.

The engineered chimeric protein was found to be stable when expressed in bacterial and human cells (Fig 2B and 2D), which allowed us to assess the functional integrity and target specificity of the protein in a series of biochemical and cell-based assays. Some of the enzymatic functions tested, including sequence-specific DNA binding and nicking, are required to mediate SSI and are shared by both parental proteins, albeit on different target DNAs. The chimera showed strong binding to DNA substrates only when they contained *oriTw* sequences, and catalyzed nicking of scDNA plasmid DNA containing *oriTw*, but not *AAVS1*. Taken together, our biochemical data showed not only that the TrwC/Rep chimeric protein maintains a functional HUH domain, but also that the redirection of this new protein is highly *oriTw*-specific since it does not recognize control substrates, highlighting the specificity of HUH proteins for their target DNAs.

Similarly, the properties provided by the C-terminal domain of the chimera, namely DNA unwinding activity and oligomerization, were expected to be similar to the parental protein Rep68. However, some differences between the parental Rep68 and the TrwC/Rep chimera were apparent. The results of the EMSA assay suggested that different oligomeric species are formed by the TrwC/Rep chimera compared to Rep68 (observe the size of the protein-DNA complexes in twin gels run in parallel, as shown in Fig 4B and 4C), and possibly also at a different protein:DNA ratio. Consistently with these apparent differences, we observed efficient but different DNA unwinding kinetics between the TrwC/Rep chimera and Rep68 (Fig 3), possibly due to differences in the number of subunits forming the oligomeric motor complex unwinding DNA. The oligomeric behaviour of the chimeric protein was studied by sedimentation equilibrium analysis, showing clearly a monomeric state with tendency to dimerize at high chimeric protein concentrations. Importantly, in the presence of substrate DNA the chimeric protein forms complexes that are larger than dimers but smaller than those formed by Rep68. The N293-TrwC domain present in the chimera has been shown to be a monomer, both in the absence and presence of substrate DNA [77]. Thus, the TrwC/Rep chimera can assemble into higher order complexes, possibly hexamers, which are different from the complexes formed by TrwC or Rep68. In summary, the combination of the relaxase domain of TrwC with the helicase domain of Rep68 seems to have produced a chimeric protein with oligomeric properties distinct from those of the parental proteins.

Our biochemical and biophysical results encouraged us to assess the ability of the TrwC/Rep chimera to function as a site-specific integrase in bacteria and human cells. The bacterial SSI assay was previously developed for TrwC, and used to determine protein and DNA requirements for SSI [43]. When the TrwC/Rep chimera was tested under the same experimental conditions, we observed that the protein was able to catalyze donor DNA SSI into an *oriTw*-containing plasmid as efficiently as TrwC (Fig 7B). In the same assay, N293-TrwC was not able to catalyse integration; thus, the Rep68 C-terminal domain of the chimera is providing some function to assist the TrwC relaxase domain in accomplishing SSI. In contrast, our attempts to detect SSI in human cells mediated by the chimera were unsuccessful (Table 2). Considering the efficiency with which TrwC and the chimera act as a site-specific integrase in bacteria (see Fig 7B), it is not surprising that we do not detect integration in the episomal assay, which lacks the sensitivity of the bacterial assay, based on positive selection of antibiotic-resistant colonies. These results correlate with a recent report showing that TrwC does not catalyse SSI into the human genome at detectable frequencies [78].

Our results indicate that the C-terminal domain of Rep68 provides a key feature to the chimeric protein to accomplish SSI in bacteria. The cross-talk between the two domains of the chimeric protein leading to SSI activity is surprising, considering that the two combined proteins are evolutionarily and biologically distant. It is unlikely that SSI is achieved through the aid of the DNA helicase activity of Rep68, since TrwC’s own DNA helicase activity was shown to be dispensable for SSI [43]. The oligomeric behavior of Rep68 is likely to play an important role in SSI of the virus into the human genome and may determine the interaction with host proteins involved in the SSI reaction, which could be evolutionarly conserved.

The ability to mediate SSI by some HUH endonucleases, such as the viral Rep protein and the bacterial relaxase TrwC, is intriguing. It is tempting to speculate that their common ancestor required integration of its genome into non-permissive genome hosts to complete its life cycle. Whether this ability is conserved in modern HUH recombinases through evolutionary selection or it is merely a remnant from a common ancestor and a consequence of the biochemical activities of the protein remains open for discussion. More specifically, while both R388-TrwC and AAV2-Rep78/68 have been shown to be necessary for SSI into their target DNA, there is no clear requirement for this function in their biological roles, i.e. plasmid conjugation and viral replication, respectively. However, it can be speculated that their SSI ability contributes to the dissemination of the parent DNA molecule, either by colonization of non-permissive hosts by the conjugative plasmid, or by vertical inheritance of the integrated viral genome.

In summary, our results suggest that HUH domains are functional units which can be linked to other domains while maintaining their biochemical activities and possibly acquiring new properties, likely illustrating the way evolution has modulated this versatile protein family. Interestingly, a recent report has revealed the high frequency of chimerism present in viral HUH Reps [79], showing incongruent evolutionary histories for their nuclease and helicase domains, which reinforces the hypothesis of modular evolution of HUH proteins.

Our approach further illustrates the utility of chimeric proteins to address the study of individual protein domain functions. Previous reports have reported swapping domains between HUH proteins in order to define the boundaries of the functional domains. AAV/goose parvovirus chimeric Rep proteins were generated by N-terminal domain swapping. This study provided biochemical evidence for the feasibility of retargeting AAV Rep DNA specificity through N-terminal domain exchange with another parvovirus (29). Our work goes one step further to show cross-talk between functional domains of two distantly related HUH proteins in order to attain new functions. Future work using Rep68 mutations, or addressing interacting partners of the chimeric and parental proteins, will shed light into the requirements needed for a HUH endonuclease to become a site-specific integrase.
